# Epidermoid Cyst of Orbit in a Newborn

**DOI:** 10.1155/2015/848427

**Published:** 2015-05-13

**Authors:** Handan Canan, Rana Altan-Yaycioglu, Nebil Bal, Birgin Törer, Bilin Çetinkaya-Çakmak, Hande Gülcan

**Affiliations:** ^1^Department of Ophthalmology, Baskent University Faculty of Medicine, 01250 Adana, Turkey; ^2^Department of Pathology, Baskent University Faculty of Medicine, 01250 Adana, Turkey; ^3^Department of Neonatology, Baskent University Faculty of Medicine, 01250 Adana, Turkey

## Abstract

A 3-day-old male newborn presented with a severe proptosis of the left eye leading to exposure keratopathy. He underwent debulking of the cyst and biopsy of the tumour and received the pathological diagnosis of epidermoid cyst of orbit. Clinicopathological features of this rare disease are discussed.

## 1. Introduction

Several orbital cystic lesions may occur in the childhood [1–3]. Cystic lesions of the orbit include cysts of the surface epithelium (dermoid and epidermoid cysts), teratomatous cyst (teratoma), neural cysts, mucocele, inflammatory cysts (parasitic cyst), lymphangioma, and rhabdomyosarcoma [1, 2, 4].

Epidermoid cyst (benign epithelial cyst) of orbit is a rare benign congenital tumor that causes proptosis in newborns. The incidence of simple epithelial cyst is uncertain. However, this cyst accounted for 8 of the 340 orbital biopsies in children from the Mayo Clinic series [3]. This abnormality is characteristically associated with a developmentally normal globe. The epidermoid cyst may exhibit rapid growth after birth, causing severe proptosis and exposure keratopathy [3].

Herein, we described a newborn that was born with severe proptosis on left orbit.

## 2. Case Report

A three-day-old male child presented with severe proptosis, which was present at birth, resembling a large mass protruding from left orbit. The child was born full term via elective cesarean section. His 26-year-old mother was healthy with normal antenatal history.

**Figure 1:**
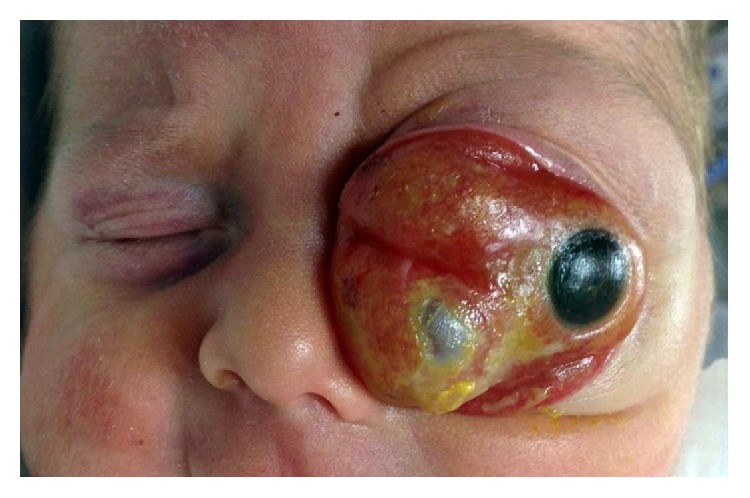
Preoperative clinical photography shows the significant proptosis and anterolateral displacement of left eye.

At presentation, his ophthalmic examination showed a large tumor in the left orbit. The tumor was protruding the globe anteriorly, preventing the occlusion of the palpebral fissure, and leading to total lagophthalmos, as well as exhibiting conjunctival chemosis and corneal haze (Figure 1). The right eye was normal.

**Figure 2:**
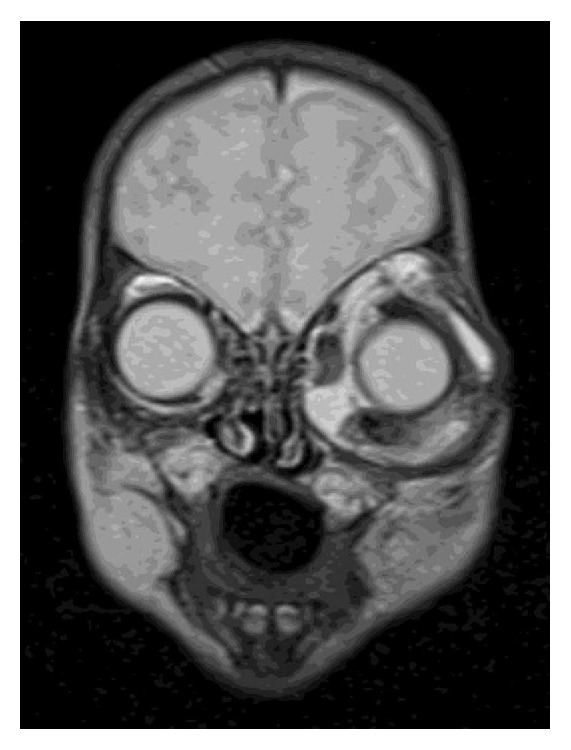
CT scan of initial presentation shows the large cystic mass was surrounding the globe and stretching the extraocular muscles in the left orbit. The right eye appears normal.

**Figure 3:**
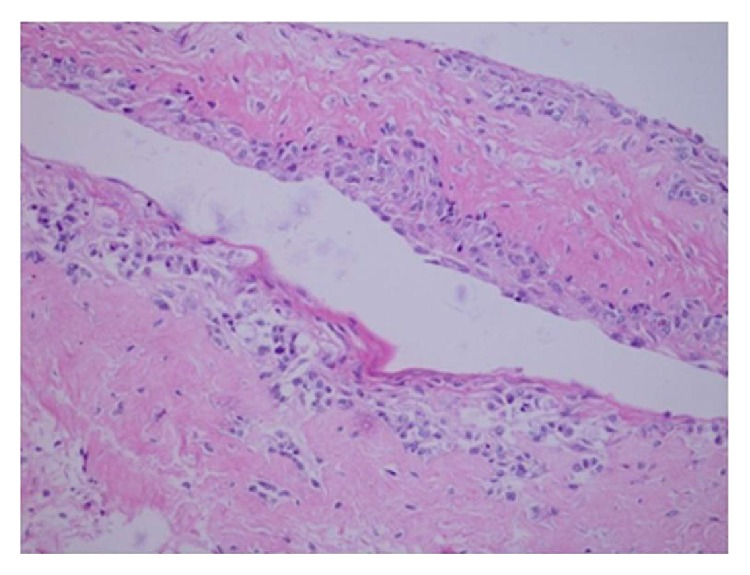
Histopathology of the cyst: cystic structure lined with squamous epithelium (hematoxylin-eosin (HE) ×200).

**Figure 4:**
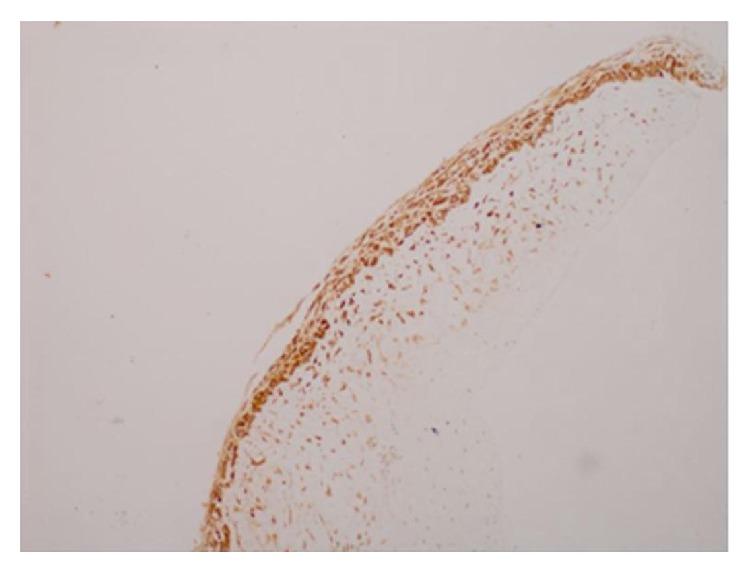
Immunohistochemical analysis of the cyst: pancytokeratin positivity at squamous epithelium (pancytokeratin ×100).

**Figure 5:**
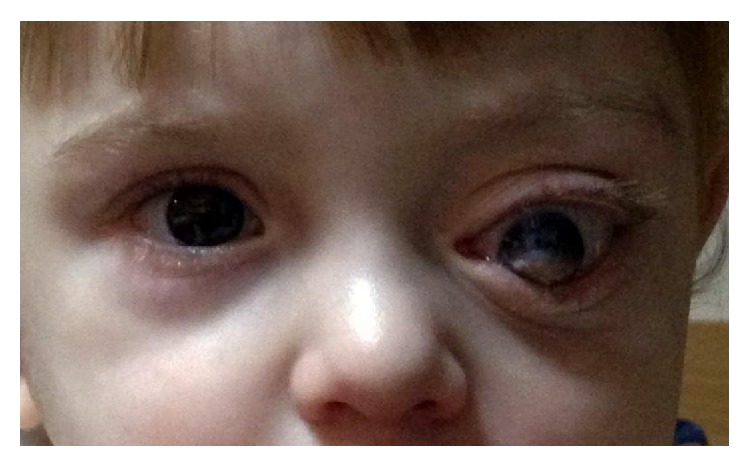
Appearance at 12 months of age.

**Figure 6:**
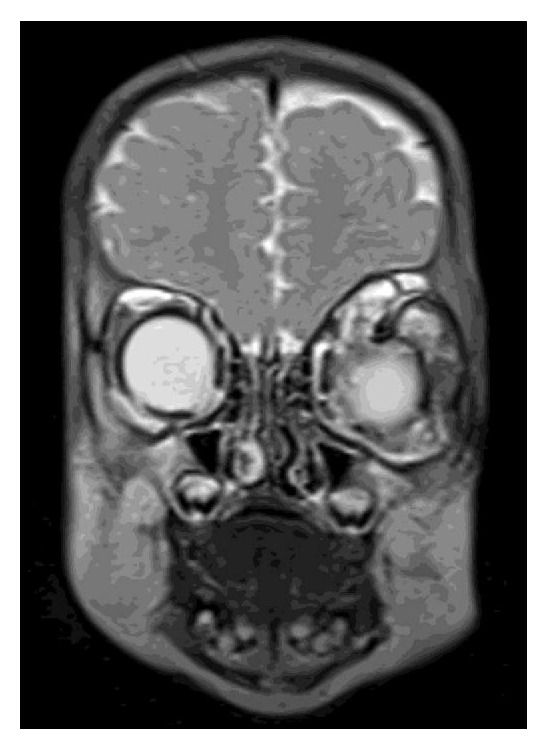
Orbital CT scan at one year of age showing the proptosis of the globe and some cystic spaces between the extraocular muscles in the left orbit.

The mass was nonpulsatile and nonreducible. Cornea was hazy because of exposure keratopathy. The child did not have any other systemic abnormality. The patient was prescribed topical moxifloxacin, nonpreserved artificial tears, and ointment. However, despite the frequent use of medication a corneal ulcer developed in two days. He had an emergent orbital computerized tomography (CT), which demonstrated a cystic lesion filling the left orbit with no apparent intracranial extension. The lesion was surrounding the globe and stretching the extraocular muscles (Figure 2). Thus, for the diagnostic purposes and to decrease the size of the tumor an operation was planned. During surgery, following nasal conjunctival peritomy, an incision in the wall of the lesion was performed and intralesional fluid was aspirated. Following debulking an incisional biopsy from the cyst wall was performed, and incision was sutured with 6-0 Vicryl. Since the eyelids were floppy and unable to close, a temporary tarsorrhaphy of the full length of eyelids was followed. Postoperatively, topical antibiotic as well as artificial tears were substituted. The cytology of the aspirated fluid revealed polymorphonuclear leukocytes and erythrocytes. No tumor cell was observed. Pathological examination of the biopsy specimen showed cystic structure lined with squamous epithelium and fibrotic wall with large areas of desquamated epithelium (Figure 3). Immunohistochemical analysis of the cyst epithelium was positive for pancytokeratin (Figure 4) and D2-40 and negative for CD68. Those immunohistochemical findings reveal the epithelial origin with squamous differentiation linings cells of the lining cyst. According to the pathological report the diagnosis was concluded as epidermoid cyst of the orbit. At the 3-month visit follow-up, the cornea healed with opacity in the central cornea and the eyelids returned to normal function with some remaining floppiness. The patient did not show up until one year of age. At this visit the globe was slightly proptotic without lagophthalmos (Figure 5). Since the child is only 1 year old, we were unable to determine the visual acuity. Central corneal opacity persisted. Control CT showed enlarged orbit compared to the right side with some fluid in intermuscular spaces (Figure 6). Since the parents were reluctant to any further surgery for the time being, we concluded to observe the patient at 3-month intervals to observe the progress of the cyst.

## 3. Discussion

Orbital cysts of the newborn are usually developmental such as dermoid and epidermoid cysts, cystic teratomas, cephaloceles, microphthalmos, and congenital cystic eye [5, 6]. The most common clinical feature of orbital cysts is mass effect, which was also the case in our patient. This mass may be as large as in our case preventing the closure of the eyelids. This exposure led to rapidly developing corneal ulcer necessitating emergent surgery. Most orbital cysts are approached surgically, with cure being affected by successful elimination of the cyst's contents and extirpation of its epithelial lining. Clinically distinguishing other benign and malignant neoplasms from cyst is difficult. Patients with possible orbital tumors should be managed by exploratory orbitotomy and excised, if possible, without damaging the eye [6, 7]. In this case, we performed the surgery to conclude the diagnosis and debulk the mass enabling the palpebral closure. In most instances, it is not possible to differentiate clinically an epidermoid cyst from other orbital cysts. Epidermoid cysts histologically have a single layer of keratinized or nonkeratinized epithelium without evidence of adnexal structures. Epidermoid cyst is usually located anteriorly in the orbit [1]. In our case the cyst was surrounding the globe pushing the globe anteriorly. In orbital cysts, the globe may be displaced or compressed due to rapid growth of the tumor, leading to vision loss as a result of perforation, collapse, secondary optic atrophy, and exposure keratopathy. Similarly in our case, the visual potential was low because of corneal opacification and ulcer, and the clinical picture deteriorated rapidly. Thus, a diagnostic biopsy and debulking surgery was performed, which might be necessary in some cases.

In conclusion, in a newborn with large orbital mass, a possible malignancy must be kept in mind. Although it is not being observed frequently, benign epidermoid cyst of the orbit is a possible diagnosis in these cases. For diagnostic purposes and preventing exposure keratopathy possibly leading to a decrease in visual functions, an immediate surgery is usually necessary.
